# Supplementary material for: Hypoxia represses pattern-triggered immune responses in Arabidopsis
Source: Plant Physiol. 2024 Aug 19;196(3):2064–77. doi: 10.1093/plphys/kiae432 (PMC11531839; doi:10.1093/plphys/kiae432)
Supplement: kiae432_Supplementary_Data [file pp.00593.2024-s10.pdf › pp.00593.2024-s10.pdf]

Plant Physiology®

Repression of pattern-triggered immune responses by hypoxia in Arabidopsis

Brian Mooney, Catherine Doorly, Melissa Mantz, Pablo Garcia, Pitter Huesgen, and Emmanuelle Graciet

Review Timeline:

Submission Date:	14-Dec-2023
Editorial Decision:	26-Jan-2024
Resubmission Received:	19-Apr-2024
Editorial Decision:	14-May-2024
Revision Received:	24-May-2024
Editorial Decision:	17-Jun-2024
Revision Received:	20-Jun-2024
Accepted:	13-Jul-2024

23-Jan-2024

Dr. Emmanuelle Graciet
Maynooth University Department of Biology
Kildare
Ireland

RE: Repression of pattern-triggered immune responses by hypoxia

Dear Dr. Emmanuelle Graciet:

Thank you for giving us the opportunity to review your manuscript for publication in the Plant Physiology. The reviewers have found the manuscript of great interest, but have raised a few concerns that will require your attention. As these concerns will entail substantial changes to the manuscript, you are likely to need additional time to address them. I must therefore reject the manuscript in its present form. Nonetheless, if you are able to address the points raised in review, I would very much welcome your resubmission of a manuscript that incorporates your responses.

In particular, I direct your attention to the comments on statistical analysis, identification of flg22-responsive genes that are mis-regulated in the a1a2 mutant, and the loading control for the immunoblot.

Please note that any resubmission will be treated as a new entry to the system and is likely to go out for review.

If you do have any queries or concerns, you are welcome to contact me in the first instance.

Thank you again for allowing Plant Physiology to review your work. We very much look forward to hearing from you soon.

Sincerely,

Editor, Plant Physiology

-- Reviewer comments:
Reviewer #1 (Comments for the Author):

Brian C. Mooney and his colleges showed a repression mechanism to PTI by hypoxia in the transcriptional level. Firstly, they hypothesized that the Arg/N-degron pathway could act in the crosstalk between abiotic/biotic stresses. Then they did transcriptome analysis, combined hypoxia/flg22 treatments further showed that hypoxia represses the flg22 transcriptional program, as well as the expression of pattern recognition receptors, MAPK signalling and callose deposition during PTI, through mechanisms that are at least partly dependent on the ERF-VIIs. The experiments were well designed and performed. This research is innovative. I have some concerns and suggestions for the authors
Major points：

1. Whether 1 or 2 h hypoxia treatment is too long for 9-day old seedlings, whether the results that you obtained is due to the depletion of energy? Whether a time gradient processing is needed at least in the later experimental validation stage?
2. Is this topic too absolute? Previous work has shown that hypoxia can also induce the expression of WRKY22 in PTI pathway. Is there a possible balancing mechanism?

Minor points:
1. The abstract needs to be concise and short.
2. Figure 2A. O2 the 2 should be subscript.
3. The font needs to be enlarged in Figure S3B-D;
4. The maker should be added in Figure S4A-B; The specific band should be marked with the detailed protein name.
5. Figure 4B erfvii should be italic.
6. The authors need to check the full text carefully to avoid the minor mistakes.

Reviewer #2 (Comments for the Author):

This work by Mooney et al aims to demonstrate the inhibitory effect acute hypoxia has on pattern triggered immune responses. Whilst mainly investigating the effect of hypoxia on the transcriptional response, the authors also consider callose deposition,

MAPK relay and expression of a number of genes, including FLS2. The authors then suggest this effect appears to be somewhat dependent on the group VII ethylene response factors, as the main actioning factors in the plant hypoxia response, which are known targets of the Arginylation branch of the N degron pathway.

Whilst a lot of work has gone into this manuscript, I think that the overall conclusions of the work are not fully supported by the data, and that many of the observations are correlative rather than the result of causation.

Major Comments

1. Lines 193-194 and Fig 1G; the authors conclude after one way ANOVA test the a1a2 mutant has a reduced level of callose deposition after flg22 treatment. However this is the incorrect test to perform on these data; a two way ANOVA looking at the effect of flg22 treatment by genotype would be more appropriate. I recommend that the authors repeat the analysis to check whether callose deposition in the a1/a2 mutant is significantly weaker.

2. Lines 222-226 and Fig 2B-C; the authors evaluate the overlap between DEGs in their flg22 RNA sequencing with a published list of DEGs after hypoxia treatment. Furthermore, a statistical overlap is then calculated to support their conclusion that the transcriptional programs of the two possess similarities. Whilst they clearly do share common DEGs, I believe this analysis has been performed incorrectly. First and foremost, the Bailey-Serres et al DEGs are based on a log2FC of 1, whereas the Mooney et al DEGs are based on a log2FC of 0.585. This might inflate the overlap between these data sets. Did the authors also consider checking the overlap with DEGs in the Mooney et al data that also have an FC value of 1? The outcome of this may also subsequently affect the number of immunity genes in fig 2C. Secondly, it is unclear how the statistically significant overlap (p>3.384e-133) was calculated? The methods refer to an online tool, however this is a black box tool with no citation nor explanation of how it works.

3. Line 251-252 Fig 2D-E; the authors identify genes which are misregulated by flg22 treatment in a1a2 relative to Col-0, many of which are hypoxia genes (HB1, HRE2, HRA1). They then show these genes are upregulated (which I take to mean hyper induced) in the a1a2flg/Col0flg comparison. From this they conclude that flg22 responsive genes are misregulated in an ERFVII-dependent manner. Direct comparison of the treated transcriptomes in this way is misrepresentative, and cannot support this conclusion. This can be seen if we consider Fig S1E which shows HB1 and HRE2 are already massively upregulated in a1a2 mock. If they are already upregulated due to the stability of ERFVIIs, their responsiveness to treatment would require that their fold change upon treatment be significantly different to the fold change upon treatment of those genes in WT. The authors should provide stronger examples of genes which are disrupted in an a1a2 dependent manner, and investigate the rate of change in responsiveness to flg22 (mock vs treated), not directly compare treated genotypes.

4. Lines 262-264 Fig S2D-G; It is unclear if the authors are showing these genes are misregulated under flg22 treatment in these genotypes. For example the increased expression of NIP2 after treatment, by FC induction, does not appear to be different to that of Col-0. Based on this I believe this data does not support crosstalk between these PTI genes and hypoxia. Whilst their regulation, based on the mock data, appears to be ERFVII dependent, their expression does not appear different relative to that of Col-0. Also, using letters to demonstrate differences in statistical tests as would improve the clarity of the figure.

5. Lines 290-291 Fig 3D; The authors conclude that combination of hypoxia and flg22 treatment enhances the induction of the 53 common genes. Have the authors considered including a figure similar to S3D here to show on a gene by gene basis that this trend is observed? It may also be more supportive of their conclusions if the significant differences were evaluated on the changes in gene expression before log transformation.

6. Lines 315-341 Fig 3H; This is an intriguing new methodology I am unfamiliar with and I think the authors have used it to great effect. To benefit readers, is there any way to assign an 'importance' metric to the suppressive power of hypoxia on flg22 responses? I also feel the novel interaction aspect is of significant interest to the community, and should possibly even be focussed on more in the text. 0.678 is a much more 'significant' score than -0.16.

7. Lines 354-357 Fig 4B; The authors conclude the dampening of PTI gene expression observed in NF vs HF conditions is diminished in an ERFVII dependent manner. To evaluate the difference in gene expression here, the authors should use a 2-way ANOVA not a 1-way ANOVA, with genotype and treatment as factors. Whilst there does appear based on the graphs to be a reduced response in erfvii, this should be backed up with appropriate statistics.

8. Fig 4E; Again this assay requires a 2 way ANOVA test to evaluate the statistical difference in callose deposition in responsiveness to treatment.

9. Lines 361-364 Fig 4C/D; This blot is not convincing of the authors conclusion that there is reduced MPK phosphorylation in the erfvii mutant upon flg22 treatment. The loading for erfvii is unequal, and in Fig S4B there is less protein loaded between genotypes. Could the authors also please indicate which band corresponds to MAPK3 and MAPK6 respectively?

10. In both the introduction and discussion the authors make no mention of other N degron substrates (ZPR2, VRN2) and how

they might be contributing to the repressive effects of hypoxia on PTI. Significantly, no mention is given to nitric oxide (which can also stabilise N degron proteins) and how it too might be a factor contributing to the results they observe (particularly as NO is a critical PTI signalling molecule). Consideration of these factors is important in the discussion.

Minor comments

Could the authors please explain why these experiments were performed in a constant light setting?

Line 116 and which

As in comment 4 above, Figures 1D and E present data which comprises a direct comparison between the flg22 treated transcriptomes of Col0 and a1a2. Plotting of the differences in FC (as you describe on line 197 for instance) would be more accurate. To support these data better consider plotting a linear regression line. Do these graphs show only the significant genes/proteins? How is the Pearson R/R2 value affected if this is fitted to all genes/proteins? Also please explain in the figure legend what DIA means.

Fig2A/ Fig3G please include the number of genes in each term, adjusted pValues alone are not informative

Lines 226-227 please give the absolute numbers of genes which were changed in the same direction (as well as the number which did not).

Please ensure any mention of the a1a2 or erfvii mutants (in body text, figure legends or figures) are italicised

Figure 4a have these data been normalised, or is the Y axis supposed to be cm? It seems odd that a root would grow only 1mm in 3 days. It also appears strange that the erfvii mutant does not appear here to be hypersensitive to hypoxia treatment.

Fig legend (Figure 4E) QuantitFication

Line 401 Flg22 doesn't appear to induce molecular hypoxia, but does induce HRE2 expression. Have the authors considering if this change in expression is observed at the protein level?

Line 491 Please soften this conclusion, this study did not use any pathogens.

Editor's comments:
In particular, I direct your attention to the comments on statistical analysis, identification of flg22-responsive genes that are mis-regulated in the a1a2 mutant, and the loading control for the immunoblot.

Response:
We have repeated the statistical analyses as requested, and have carried out additional biological replicates, where we felt that variation was a problem. That allowed us to draw conclusions from a larger number of biological replicates for the MPK immunoblot, as well as RT-qPCRs with combined hypoxia/flg22. We also provide a better set of immunoblots and loading controls in the revised version of our manuscript.

All reviewers requests have been addressed, as outlined in detail below.

Reviewer #1 (Comments for the Author):

Brian C. Mooney and his colleagues showed a repression mechanism to PTI by hypoxia in the transcriptional level. Firstly, they hypothesized that the Arg/N-degron pathway could act in the crosstalk between abiotic/biotic stresses. Then they did transcriptome analysis, combined hypoxia/flg22 treatments further showed that hypoxia represses the flg22 transcriptional program, as well as the expression of pattern recognition receptors, MAPK signalling and callose deposition during PTI, through mechanisms that are at least partly dependent on the ERF-VIIs. The experiments were well designed and performed. This research is innovative. I have some concerns and suggestions for the authors.

Major points ：
1. Whether 1 or 2 h hypoxia treatment is too long for 9-day old seedlings, whether the results that you obtained is due to the depletion of energy? Whether a time gradient processing is needed at least in the later experimental validation stage?

Response:
The 1-hr hypoxia treatment used here corresponds to the early stages of the hypoxia response program, based on (i) the induction of *HRE1* and *HRE2* (two of the ERFVIIs) around 1 hr of hypoxia; (Licausi et al., 2010), (ii) the timing of RAP2.12 (another ERFVII) translocation into the nucleus around 3 hrs after the onset of hypoxia (Kosmacz et al., 2015), (iii) several transcriptomic datasets generated using Arabidopsis seedlings at different time points after the onset of hypoxia, and which show that 1-2 hrs is still within early changes in genes expression in response to hypoxia (e.g. (Liu et al., 2005; Mustroph et al., 2009; Lee and Bailey-Serres, 2019)).

2. Is this topic too absolute? Previous work has shown that hypoxia can also induce the expression of WRKY22 in PTI pathway. Is there a possible balancing mechanism?

Response:

We thank the reviewer for this comment and making us aware of the Hsu et al. (2013) (Hsu et al., 2013) paper describing the regulation of WRKY22 by dark submergence treatment and its role in response to pathogens. The experimental conditions are very different in Hsu et al (2013) and in our manuscript, which makes direct comparisons between their data and ours very difficult. For example, Hsu et al. used submergence in the dark for longer periods of time, while we use hypoxia under light conditions. Also, they use pathogens, while we use the PAMP flg22, and finally they conduct the pathogen inoculation after submergence (during reoxygenation), whereas we combine at the same time hypoxia and flg22. We are unsure what the reviewer meant by 'balancing mechanism', but could envision it in 2 different conceptual ways:

- a 'balancing mechanism' may be needed to ensure that immunity is up-regulated when plants are experiencing submergence stress, because such conditions could favor pathogen infection. This is possible, but our data does not allow us to draw conclusions on such mechanisms. This was also not the aim of our manuscript.
- the 'balancing mechanism' could refer to the need to return to normal homeostasis after submergence and reoxygenation. Again, we cannot answer this question based on our experiments, and this was not the aim of our study.

Nevertheless, we have compared our list of 384 DEGs common to the Col-0 f/m and 2HS/2NS datasets with lists of DEGs in a *wrky22* mutant or of genes whose promoter is bound by WRKY22. We only retrieved 3 genes in common. This is in line with the fact that *WRKY22* expression was not differentially expressed in the Col-0 f/m dataset. Furthermore, we checked the expression of *WRKY22* in our RNA-seq dataset obtained under combined hypoxia/flg22 (HF) conditions, and found that *WRKY22* expression was up ($log_2FC=3.29$). Interestingly, 11 (out of 45) genes bound by WRKY22 were then identified in our RNA-seq HF dataset. We have included a short paragraph in our revised Discussion.

Minor points:

1. The abstract needs to be concise and short.

Response:

We have shortened the abstract and made it more concise.

2. Figure 2A. O2 the 2 should be subscript.

Response:

We have corrected this mistake.

3. The font needs to be enlarged in Figure S3B-D;

Response:

Font size was increased.

4. The marker should be added in Figure S4A-B; The specific band should be marked with the detailed protein name.

Response:
We have added the requested labelling.

5. Figure 4B erfvii should be italic.

Response:
This figure panel changed.

6. The authors need to check the full text carefully to avoid the minor mistakes.

Response:
We have done so before submitting this revised version.

Reviewer #2 (Comments for the Author):

This work by Mooney et al aims to demonstrate the inhibitory effect acute hypoxia has on pattern triggered immune responses. Whilst mainly investigating the effect of hypoxia on the transcriptional response, the authors also consider callose deposition, MAPK relay and expression of a number of genes, including FLS2. The authors then suggest this effect appears to be somewhat dependent on the group VII ethylene response factors, as the main actioning factors in the plant hypoxia response, which are known targets of the Arginylation branch of the N degron pathway.

Whilst a lot of work has gone into this manuscript, I think that the overall conclusions of the work are not fully supported by the data, and that many of the observations are correlative rather than the result of causation.

Major Comments

1. Lines 193-194 and Fig 1G; the authors conclude after one way ANOVA test the a1a2 mutant has a reduced level of callose deposition after flg22 treatment. However this is the incorrect test to perform on these data; a two way ANOVA looking at the effect of flg22 treatment by genotype would be more appropriate. I recommend that the authors repeat the analysis to check whether callose deposition in the a1/a2 mutant is significantly weaker.

Response:
We thank the reviewer for this comment, and similar ones below, all of which have been addressed by using a 2-way ANOVA followed by a Tukey *post-hoc* test. The re-analysis of the data in Figure 1G shows that row and column effects, as well as interaction between genotypes and treatment are all significant. The comparison of Col-0 flg22 to a1a2 flg22 is also significant. To address another comment made in point 4 below, we have also

changed the presentation of the results of statistical tests to use the Compact Letter Display format. Below is a copy of the new panel 1G.

Source of Variation	% of total variation	P value	P value summary	Significant?
Interaction	1.862	0.0009	***	Yes
Genotype	1.560	0.0022	**	Yes
Treatment	69.95	<0.0001	****	Yes

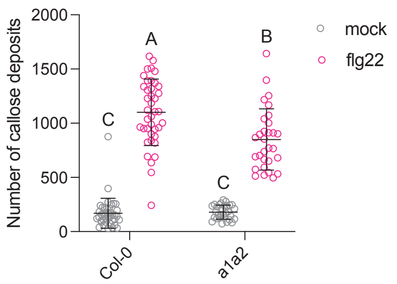

2. Lines 222-226 and Fig 2B-C; the authors evaluate the overlap between DEGs in their flg22 RNA sequencing with a published list of DEGs after hypoxia treatment. Furthermore, a statistical overlap is then calculated to support their conclusion that the transcriptional programs of the two possess similarities. Whilst they clearly do share common DEGs, I believe this analysis has been performed incorrectly. First and foremost, the Bailey-Serres et al DEGs are based on a log_2FC of 1, whereas the Mooney et al DEGs are based on a log_2FC of 0.585. This might inflate the overlap between these data sets. Did the authors also consider checking the overlap with DEGs in the Mooney et al data that also have an FC value of 1? The outcome of this may also subsequently affect the number of immunity genes in fig 2C. Secondly, it is unclear how the statistically significant overlap (p>3.384e-133) was calculated? The methods refer to an online tool, however this is a black box tool with no citation nor explanation of how it works.

Response:

a. The choice of cut-offs in transcriptomics experiments is arbitrary, with some considering that the only cut-off needed is on the adjusted *p*-values because small but statistically significant fold changes can be biologically relevant, while others consider that in addition to the adjusted *p*-value cut-off, a cut-off on the fold-change is necessary. In this case, the most common cut-offs would be fold changes of 2 or 1.5. For our analyses, in addition to applying a cut-off of adjusted *p*-value<0.05, we also chose to apply a cut-off of 1.5 on the fold-change (or $|log_2(FC)|>0.585$) because comparison with other flg22 response datasets indicated that genes with $|log_2(FC)|$ values between 1 and 0.585 were relevant to flg22 transcriptional responses. The data presented in Figures 1 and S1 validate our datasets and cut-offs used.

Because our datasets and those generated by Lee et al originate from different experimental conditions, applying the same cut-offs to these 2 different datasets is an arbitrary decision. To address the reviewer's concern, we have nevertheless repeated the analyses using the proposed cut-offs of |log2(FC)|>1 and adj. *p*-value<0.05. Our findings remain the same: GO analysis of Col-0 flg/m still retrieves an enrichment for

genes associated with hypoxia response and the overlap between Col-0 flg/m and the 2HS/2NS datasets obtained under hypoxia remain statistically significant. In fact, for the latter, when comparing the Col-0 flg/m and 2HS/2NS datasets, the overlap is proportionally larger than when we used a |log2(FC)|>0.585 cut-off for the Col-0 flg/m dataset, and hence the point we are making – that there is an overlap between the flg22 and hypoxia response programs – is in fact reinforced when using the more stringent cut-offs.

We decided to retain our initial analysis with |log2(FC)|>0.585 in the manuscript, but now provide additional analyses with log2(FC)|>1 in new Supplementary Figure S3.

b. We agree that the online tool to calculate the *p*-values when comparing gene lists and assessing statistical significance of the overlap is not clearly explained. It is worth noting though, that it has been used in other publications such as (https://www.ncbi.nlm.nih.gov/pmc/articles/PMC4528312/); https://doi.org/10.1093/nargab/lqac001; https://doi.org/10.1016/j.ebiom.2016.10.026) We have nevertheless repeated all the calculations using 2x2 contingency tables and Chi square tests. For all calculations (i.e. done for Fig.2B and Fig. S2B), we found similar results and the conclusions on statistical significance of the overlap are unchanged. We have edited the text and Materials and Methods accordingly.

3. Line 251-252 Fig 2D-E; the authors identify genes which are misregulated by flg22 treatment in a1a2 relative to Col-0, many of which are hypoxia genes (HB1, HRE2, HRA1). They then show these genes are upregulated (which I take to mean hyper induced) in the a1a2flg/Col0flg comparison. From this they conclude that flg22 responsive genes are misregulated in an ERFVII-dependent manner. Direct comparison of the treated transcriptomes in this way is misrepresentative, and cannot support this conclusion. This can be seen if we consider Fig S1E which shows HB1 and HRE2 are already massively upregulated in a1a2 mock. If they are already upregulated due to the stability of ERFVIIs, their responsiveness to treatment would require that their fold change upon treatment be significantly different to the fold change upon treatment of those genes in WT. The authors should provide stronger examples of genes which are disrupted in an a1a2 dependent manner, and investigate the rate of change in responsiveness to flg22 (mock vs treated), not directly compare treated genotypes.

<u>Response:</u>
We thank the reviewer for this comment. Upon careful consideration of the comment, we agree with the reviewer that this analysis is probably not the most direct one to assess whether the expression of some flg22 response genes could be ERFVII dependent (or not). We have therefore removed the panels in Figure 2D/E/F and instead considered genes whose expression was:

- responsive to hypoxia in the 2HS/2NS dataset from Lee et al (2019)
- AND responsive to flg22 in our Col-0 flg/m dataset
- AND either (i) respond more strongly in a1a2 flg/m compared Col-0 flg/m; or (ii) are already significantly up-regulated in mock-treated a1a2 (likely because of the ERFVII accumulation).

We then tested the expression of several of these genes by RT-qPCR in Col-0 and in the *erfVII* mutant. The results indicate that the regulation of these genes by flg22 is

not ERFVII dependent. The data is now provided in the revised Figure 2D and we have modified our conclusions and discussion accordingly

4. Lines 262-264 Fig S2D-G; It is unclear if the authors are showing these genes are misregulated under flg22 treatment in these genotypes. For example the increased expression of NIP2 after treatment, by FC induction, does not appear to be different to that of Col-0. Based on this I believe this data does not support crosstalk between these PTI genes and hypoxia. Whilst their regulation, based on the mock data, appears to be ERFVII dependent, their expression does not appear different relative to that of Col-0. Also, using letters to demonstrate differences in statistical tests as would improve the clarity of the figure.

Response:
Considering the new experiments we conducted and the changes made to address point 3 of this reviewer, we believe that the data presented in Figure S2 is no longer relevant. This data has therefore been removed from the manuscript. Our conclusions are now drawn from the new data presented in the revised Fig. 2 and is in agreement with the reviewer's point that the differences are likely not ERFVII dependent.

The reviewer suggests to use the Compact Letter Display format to show the results of statistical tests. We have switched to this format in all relevant figure panels.

5. Lines 290-291 Fig 3D; The authors conclude that combination of hypoxia and flg22 treatment enhances the induction of the 53 common genes. Have the authors considered including a figure similar to S3D here to show on a gene by gene basis that this trend is observed? It may also be more supportive of their conclusions if the significant differences were evaluated on the changes in gene expression before log transformation.

Response:
We have addressed the reviewer's comments in 2 ways:
- we have added a new panel 3D which shows the $\log_2$(FC) values for all 53 genes as a heatmap
- we have also changed the previous panel 3F (now 3G) so that the readers may follow each of the 2 genes expression changes. One of the genes has filled symbols, and the other one open symbols.

6. Lines 315-341 Fig 3H; This is an intriguing new methodology I am unfamiliar with and I think the authors have used it to great effect. To benefit readers, is there any way to assign an 'importance' metric to the suppressive power of hypoxia on flg22 responses? I also feel the novel interaction aspect is of significant interest to the community, and should possibly even be focussed on more in the text. 0.678 is a much more 'significant' score than -0.16.

Response:
We have addressed the reviewer's comment to contextualize the values by presenting in more detail a comparison with the average and highest/lowest score values obtained by Tan et al (2023) when combining stress in Marchantia (Tan et al., 2023). We agree that

this further supports the relevance of the interaction score obtained for the novel interaction score with down-regulated genes upon combined hypoxia/flg22 treatment. We have retained the points made in the discussion, as presented in the first version of the manuscript. For ease of review, we include below a table with relevant values (Table 1).

Table 1: Comparison of scores in Marchantia and in our hypoxia/flg22 RNA-seq dataset

	Marchantia		Hypoxia/flg22	
	Up-regulated	Down-regulated	Up-regulated	Down-regulated
Similarity score	Highest: 0.27<br>Median: 0.1<br>Average: 0.12	Highest: 0.26<br>Median: 0.075<br>Average: 0.097	0.031	0.0061
Novel interaction score	Highest:0.48<br>Median: 0.27<br>Average: 0.29	Highest: 0.56<br>Median: 0.30<br>Average: 0.29	0.241	0.678
Suppression score (only negative scores considered for Marchantia)	Lowest: -0.65<br>Median: -0.3<br>Average: -0.29	Lowest: -0.42<br>Median: -0.1<br>Average: -0.15	-0.031	-0.160

7. Lines 354-357 Fig 4B; The authors conclude the dampening of PTI gene expression observed in NF vs HF conditions is diminished in an ERFVII dependent manner. To evaluate the difference in gene expression here, the authors should use a 2-way ANOVA not a 1-way ANOVA, with genotype and treatment as factors. Whilst there does appear based on the graphs to be a reduced response in erfvii, this should be backed up with appropriate statistics.

Response:
We have now performed 2-way ANOVAs, and used the CLD format to present the data. Because of the variation between biological replicates, we also decided to add one more biological replicates to these panels to increase the robustness of the statistical analysis and of our conclusions. Our results now show that the differences are mostly ERFVII independent.

8. Fig 4E; Again this assay requires a 2 way ANOVA test to evaluate the statistical difference in callose deposition in responsiveness to treatment.

Response:
We have performed a 2-way ANOVA for Figure 4E and have found the same results as before. The graph has been changed, and CLD format has been used to indicate the results of the 2-way ANOVA with post-hoc Tukey tests.

9. Lines 361-364 Fig 4C/D; This blot is not convincing of the authors conclusion that there is reduced MPK phosphorylation in the erfvii mutant upon flg22 treatment. The loading for erfvii is unequal, and in Fig S4B there is less protein loaded between genotypes. Could the authors also please indicate which band corresponds to MAPK3

and MAPK6 respectively?

Response:
We have added labels to indicate which bands correspond to MPK6 (upper band) and MPK3 (lower band). We agree with the reviewer that the immunoblot for *erfVII* is not optimum, because the Ponceau shows a mild decrease in signal. However, the data that was provided in Fig. 4D was generated by normalizing the MPK6/3 signal intensity for the corresponding Ponceau signals in each lane. Hence, the data in Fig. 4D corrects for that problem.
Nevertheless, because of the large error bar after normalization and relative quantification of HF to NF signals, we decided to perform additional biological replicates for the *erfVII* (and Col-0 as a control) immunoblots and increase the robustness of our data. With the additional replicates carried out, our conclusion is that ERFVIIs do not play a role in the reduced MPK signalling under HF compared to NF.

10. In both the introduction and discussion the authors make no mention of other N degron substrates (ZPR2, VRN2) and how they might be contributing to the repressive effects of hypoxia on PTI. Significantly, no mention is given to nitric oxide (which can also stabilise N degron proteins) and how it too might be a factor contributing to the results they observe (particularly as NO is a critical PTI signalling molecule). Consideration of these factors is important in the discussion.

Response:
We have now included a mention of *ZPR2* and *VRN2* in both the Introduction and the Discussion, as well as the potential roles of NO in response to combined hypoxia/flg22 (in the Discussion).

Minor comments

Could the authors please explain why these experiments were performed in a constant light setting?

We have grown plants under continuous light settings in order to ensure that Col-0 and Arg/N-degron mutants such as *a1a2* would be developmentally indistinguishable. In other light conditions, such as short days, and in some cases long days, the *a1a2* mutant grows more slowly than the wild type, which could affect its response to flg22. For experiments involving hypoxia, we kept the plants under the same light conditions, to avoid gene expression changes triggered by dark treatment. The latter would have confounded our study, by triggering a triple stress (dark/hypoxia/flg22), as opposed to the double stress (hypoxia/flg22) we wished to focus on.

As in comment 4 above, Figures 1D and E present data which comprises a direct comparison between the flg22 treated transcriptomes of Col0 and a1a2. Plotting of the differences in FC (as you describe on line 197 for instance) would be more accurate. To support these data better consider plotting a linear regression line. Do these graphs

show only the significant genes/proteins? How is the Pearson R/R2 value affected if this is fitted to all genes/proteins? Also please explain in the figure legend what DIA means.

The graphs presented in Fig.1D/E only present significant genes/ proteins. We believe that this is a more rigorous analysis than without any cut-offs on statistical significance. We have considered carefully the reviewer's suggestion and are a bit unsure about what was meant by 'plotting differences in FC', because we are showing log2(FC). We think that maybe the reviewer is asking whether it might be better to plot the differences of Col-0 flg/m and *a1a2* flg/m. Our aim was to determine protein accumulation differences between *a1a2* and the wild-type, because of the possibility that *a1a2* differences relative to the wild type may be linked to a deficiency in Arg/N-degron mediated protein degradation. Also, in our proteomics datasets, genotype differences contributed more than the treatment to differential protein accumulation. Altogether, we concluded that it was best to keep Figure 1D/E as in the initial submission of our manuscript. We have however added the linear regression line as requested by the reviewer, and removed mention of Pearson r in the manuscript. We have now also indicated that DIA stands for Data Independent Acquisition mode.

Fig2A/ Fig3G please include the number of genes in each term, adjusted pValues alone are not informative

For Fig. 2A, the number of genes in each GO category are presented in Supplemental Dataset 1. For Fig. 3H in the revised manuscript, the number of genes are presented in Supplemental Dataset 5. We now specifically indicate that the complete list of GO categories and number of DEGs in each GO category can be found in these Supplemental Datasets.

Lines 226-227 please give the absolute numbers of genes which were changed in the same direction (as well as the number which did not).

We now state that 55 out of 59 DEGs have the same directionality of gene expression change.

Please ensure any mention of the a1a2 or erfvii mutants (in body text, figure legends or figures) are italicised

We have italicized *a1a2* and *erfVII* as requested.

Figure 4a have these data been normalised, or is the Y axis supposed to be cm? It seems odd that a root would grow only 1mm in 3 days. It also appears strange that the erfvii mutant does not appear here to be hypersensitive to hypoxia treatment.

We apologize for mis-labelling the y axis of Fig. 4A. The data presented is indeed relative to the root length in untreated conditions (NM). This has been corrected. We are also

surprised that the hypersensitivity of the *erfVII* mutant is not observed. However, the fact that the wild type Col-0 behaved as expected under hypoxia suggested that the treatment was sufficient to monitor effects of hypoxia on PTI.

Fig legend (Figure 4E) QuantitFication

We have made the requested change from 'quantitation' to 'quantification'.

Line 401 Flg22 doesn't appear to induce molecular hypoxia, but does induce HRE2 expression. Have the authors considering if this change in expression is observed at the protein level?

We have not checked the HRE2 protein levels.

Line 491 Please soften this conclusion, this study did not use any pathogens.

We have softened our conclusion, as requested by the reviewer.

References

Hsu FC, Chou MY, Chou SJ, Li YR, Peng HP, Shih MC (2013) Submergence confers immunity mediated by the WRKY22 transcription factor in Arabidopsis. Plant Cell **25:** 2699-2713

Kosmacz M, Parlanti S, Schwarzlander M, Kragler F, Licausi F, Van Dongen JT (2015) The stability and nuclear localization of the transcription factor RAP2.12 are dynamically regulated by oxygen concentration. Plant Cell Environ **38:** 1094-1103

Lee TA, Bailey-Serres J (2019) Integrative Analysis from the Epigenome to Translatome Uncovers Patterns of Dominant Nuclear Regulation during Transient Stress. Plant Cell **31:** 2573-2595

Licausi F, van Dongen JT, Giuntoli B, Novi G, Santaniello A, Geigenberger P, Perata P (2010) HRE1 and HRE2, two hypoxia-inducible ethylene response factors, affect anaerobic responses in Arabidopsis thaliana. Plant J **62:** 302-315

Liu F, Vantoai T, Moy LP, Bock G, Linford LD, Quackenbush J (2005) Global transcription profiling reveals comprehensive insights into hypoxic response in Arabidopsis. Plant Physiol **137:** 1115-1129

Mustroph A, Zanetti ME, Jang CJ, Holtan HE, Repetti PP, Galbraith DW, Girke T, Bailey-Serres J (2009) Profiling translatomes of discrete cell populations resolves altered cellular priorities during hypoxia in Arabidopsis. Proc Natl Acad Sci U S A **106:** 18843-18848

Tan QW, Lim PK, Chen Z, Pasha A, Provart N, Arend M, Nikoloski Z, Mutwil M (2023) Cross-stress gene expression atlas of Marchantia polymorpha reveals the hierarchy and regulatory principles of abiotic stress responses. Nat Commun **14:** 986

14-May-2024

Dr. Emmanuelle Graciet
Maynooth University Department of Biology
Kildare
Ireland

RE: Repression of pattern-triggered immune responses by hypoxia

Dear Dr. Graciet:

We have received reviews of your manuscript, which you may view at the end of this letter. On the basis of the advice received and my own evaluation, I believe that, pending revision, this article may ultimately be acceptable for publication in Plant Physiology.

I draw your attention to the comments by Reviewer #1 regarding the activation of MPKs in response to hypoxia, which is previously described as increasing and peaking within 2 hours of exposure to oxygen deprived conditions (Plant Molecular Biology 78 (2012): 109-122). Also, consider the comments from Reviewer #3 on the comparison of the transcriptional response of a1a2 to that of wild type following flg22 treatment. As well as further discussions on the significance of the repression of PTI, including the reduction in callose deposition, by hypoxia.

When you are ready to submit your revision, click the below link and find the red arrow under your Author Tasks heading for this revision submission.

If you want to visit this version of the manuscript, you may click the link below and then click on the folder that reads "Post Decision Manuscripts".

Link Not Available

When uploading the revised version of this article, please be sure to include as complete an answer as possible to the referees' criticisms during the first round of reviews in the "response to reviewers" section.

Plant Physiology considers a revision for up to 60 days after a manuscript has been provisionally accepted; limited extension of this timeframe is at the discretion of the Monitoring Editor. A reminder notice will be sent to you 2 weeks before the deadline, at which time you may choose to contact the editorial office to request an extension; if so, please provide a justification for your request. After the expiration of this deadline, the article would need to be submitted as a resubmission and would be handled as such.

Thank you very much for giving us an opportunity to review this work. I look forward to receiving the next version.

Sincerely,

Editor, Plant Physiology

Want to add this revision deadline to your calendar? Click below!

-- Reviewer comments:
Reviewer #1 (Comments for the Author):

The current version has seen significant improvements and enhancements, but I still have a few minor issues to address before accepting this article.The following are my suggestions：

1. It is suggested to add words like "plant" or "Arabidopsis" in the title.
2. In Figure 4C, after 2 hours of hypoxia treatment in other studies (Plant Mol Biol (2012) 78:109-122), MPK6 was found to be activated. Could you discuss this result?
3. In Figure S5A-B, the molecular weight of proteins is not clearly labeled, and it's not clear which protein is being referred to as

the target. Additionally, the quantification of the target protein under different treatments should be done using ImageJ to increase the readability of the article.

Reviewer #3 (Comments for the Author):

This article written by Mooney & Doorly et al seeks to investigate the overlap between hypoxia and flg22-triggered PTI responses in Arabidopsis. I reviewed the first submission of this article in January 2024 and thank the authors for their thoughtful responses to my initial concerns and queries. A lot of work has gone into addressing my comments. Nonetheless, I do have some further comments the authors might consider addressing.

Line 147: Did the authors consider performing GO term enrichment analysis on these 700 and 507 genes to identify the differences in the transcriptional responses of a1a2 and Col-0? This seems (at least to me) a more intuitive way to compare the a1a2 and col0 flg22-treated transcriptomes- particularly as the way the authors later perform this comparison (line 152-168) is slightly biased towards hypoxia genes (line 158) anyway given their constitutive upregulation in a1a2.

Line 217-221, Figure S1E: Is HB1 here actually differentially expressed? Given the context, the a1a2 data here is not telling us anything. Have the authors considered showing only Col-0 expression data, differences to which I suspect are being skewed due to the massive upregulation of these hypoxia markers in a1a2. Also please consider showing data for all genes mentioned in the text body (EDS1 and PDF4 for instance).

Line 315: the hypoxia response program represses the (downregulated) flg22 transcriptional response. The suppressive metric of -0.031 for upregulated genes seems arbitrary, particularly when we consider the median repressive score (for upregulated genes) in Tan et al. was 0.3.

I would personally consider concluding less strongly (e.g. our analysis suggests that...), particularly as the concluding remark to this passage is somewhat open ended "the repression of down-regulated genes by hypoxia could be of relevance", and the significance to the biology of this observation is also not immediately clear.

Line 350: Authors conclude that the erfvii mutant was more severely affected by the combined stress compared to HM and NF conditions - however based on the statistical differences displayed in figure 4A this is only true for HM. Similar to erfvii, the Col-0 response to NF is also significantly different to the HF condition.

Line 411-416: Given that it's the title of this paper, could the authors comment on the biological significance of the repression if PTI by hypoxia in the discussion? Intuitively this might be to do with severity of each stress respectively, i.e hypoxia takes 'priority' as this can kill the plant much faster than any pathogen might. 304 genes no longer being differentially expressed is certainly a remarkable observation.

Line 35: degradation accumulation
Line 122: "suggested a transcriptional link between hypoxia responses and PTI.
Line 170: ATE1 should be italicised
Line 189: SAG21 should be italicised
Line 208: Our data suggest a potential connection between hypoxia and flg22 responses. The RNA-seq analysis also revealed...
Line 214: in across
Line 219: or and
Line 229: (e.g. LBD41 or HRE2; Fig. S1E)
Line 230: or and
Line 231: Define PRR, I didn't see it beforehand
Line 332: aspects of the flg22 response.
Line 333: i.e these genes which no longer
Line 337: Not sure if I missed it, define EFR
Line 346: Either Both flg22 (NF) or and hypoxia...
Line 435-442: Please italicise all mentions of WRKY22 when the gene is being referred to and not the protein.
Figure 4B: Italicise gene names (EFR, FLS2, MYB51, CYP83B1)

Reviewer #1 (Comments for the Author):

The current version has seen significant improvements and enhancements, but I still have a few minor issues to address before accepting this article. The following are my suggestions ：

1. It is suggested to add words like "plant" or "Arabidopsis" in the title

Response

We have changed the title to "**Repression of pattern-triggered immune responses by hypoxia in Arabidopsis**".

2. In Figure 4C, after 2 hours of hypoxia treatment in other studies (Plant Mol Biol (2012) 78:109-122), MPK6 was found to be activated. Could you discuss this result?

Response

We have carefully read the publication indicated by the reviewer. It shows that MPK3/4/6 are activated in a transient manner upon hypoxia in 10-day old seedlings. These results were obtained for time points comprised between 30 minutes and 8 hours of hypoxia using in-gel kinase activity with radioactive ATP. The authors show a mild induction of MPK activity at 30 minutes, with a peak at 2 hours and then a decrease in MPK activity at 8 hours.

These experiments differ from ours, which could explain why we do not detect an increase in phosphorylated MPKs after 30 minutes of hypoxia:
- the use of a radioactive assay to detect MPK activity is likely to be more sensitive than the immunoblot approach we used. This may have allowed Chang et al. to detect the weak up-regulation of MPK activity at 30 minutes of hypoxia.
- the 30-minute time point we have used does not correspond to the peak of MPK activity under hypoxia. Combined with the use of immunoblots instead of a radioactive assay, we may not be able to detect an increase in phosphorylated MPK at this time point.

We therefore believe that our data is not conflicting with those of Chang *et al*. Our use of a 30-minute time point for our experiments was justified because of the faster induction of flg22-induced activation of MPKs.

We have modified our Results section and cite Chang *et al*. as follows:
"As expected, in Col-0, phosphorylation of MPK3 and MPK6 was induced after 30 min of flg22 treatment under normoxia (NF). We did not observe a detectable accumulation of phosphorylated MPKs by immunoblot after a 30-min hypoxia treatment alone, likely because MPK3/6 activity peaks at 2 hrs of hypoxia and is only weakly activated at 30 min, as shown using more sensitive radioactive in-gel kinase assays (Chang et al., 2012). Notably, the level of phosphorylated MPK3/6 was reduced under combined hypoxia/flg22 treatment compared to flg22 treatment alone. Similar effects were observed in the *erfVII* mutant (Fig. 4C, Fig. 4D and Fig. S5B)."

3. In Figure S5A-B, the molecular weight of proteins is not clearly labeled, and it's not clear which protein is being referred to as the target. Additionally, the quantification of the target protein under different treatments should be done using ImageJ to increase the readability of the article.

Response
In Figure S5A-B, the molecular weights of the marker proteins in the pre-stained ladder are now indicated. We have also labelled the proteins of interest using arrows of different colors. The figure legend now also includes these details, as well as the expected molecular weight of each of the proteins of interest.

For the MPK immunoblot, we have quantified the signal intensity for both MPKs together across 9 and 6 biological replicates for Col-0 and *erfVII*, respectively, and had presented the data as the signal ratio of HF/NF in Fig. 4D. This was done using Image J, which we have now clearly indicated in the Materials and Methods. We are unsure about what the reviewer is requesting, but to be thorough, we are now indicating in Fig. S5A-B the quantification of Ponceau-normalized intensity values for the proteins/bands of interest.

Reviewer #3 (Comments for the Author):

This article written by Mooney & Doorly et al seeks to investigate the overlap between hypoxia and flg22-triggered PTI responses in Arabidopsis. I reviewed the first submission of this article in January 2024 and thank the authors for their thoughtful responses to my initial concerns and queries. A lot of work has gone into addressing my comments. Nonetheless, I do have some further comments the authors might consider addressing.

Line 147: Did the authors consider performing GO term enrichment analysis on these 700 and 507 genes to identify the differences in the transcriptional responses of a1a2 and Col-0? This seems (at least to me) a more intuitive way to compare the a1a2 and col0 flg22-treated transcriptomes- particularly as the way the authors later perform this comparison (line 152-168) is slightly biased towards hypoxia genes (line 158) anyway given their constitutive upregulation in a1a2.

Response
We had indeed from the beginning performed the suggested GO analysis with the 700 and 507 DEGs specific to a1a2 flg/m and Col-0 flg/m, respectively, but neither had GO biological process categories enriched. This indicates that these genotype-specific DEGs in response to flg22 comprise genes from a range of biological processes and not from specific pathways that may be disrupted or regulated in one particular genotype. Because of the absence of enriched GO categories, we had not deemed it relevant to mention these results in the 2 previous versions of the manuscript.

We have added the following sentence: "Neither of these sets of DEGs are enriched for specific GO biological process categories, suggesting that they comprise genes associated with a wide range of functions."

Line 217-221, Figure S1E: Is HB1 here actually differentially expressed? Given the context, the a1a2 data here is not telling us anything. Have the authors considered showing only Col-0 expression data, differences to which I suspect are being skewed due to the massive upregulation of these hypoxia markers in a1a2. Also please consider showing data for all genes mentioned in the text body (EDS1 and PDF4 for instance).

Response

We agree with the reviewer that showing the data for the *a1a2* mutant is not necessary to support the point made in the manuscript (i.e. that hypoxia response genes are also differentially expressed during the onset of flg22 transcriptional response in wild-type Col-0 plants). We wish to emphasize that the expression of these genes is identified as differentially expressed in our RNA-seq datasets using the cut-offs indicated ($|\log_2 FC|>0.585$ and adj. p-value < 0.05). We have followed the reviewer's suggestions and have:

- removed the *a1a2* data
- added the data for *AlaAT1*, *PAD4* and *EDS1*

In doing so, the data was then best placed into Fig. S2, so this figure was changed and all panels in Fig. S1 were also re-arranged accordingly. One panel (previous S1F) was changed to show only Col-0 mock and *a1a2* mock data to support our point that hypoxia response genes are up-regulated in *a1a2* compared to Col-0 in the absence of flg22, as expected.

Line 315: the hypoxia response program represses the (downregulated) flg22 transcriptional response. The suppressive metric of -0.031 for upregulated genes seems arbitrary, particularly when we consider the median repressive score (for upregulated genes) in Tan et al. was 0.3. I would personally consider concluding less strongly (e.g. our analysis suggests that...), particularly as the concluding remark to this passage is somewhat open ended "the repression of down-regulated genes by hypoxia could be of relevance", and the significance to the biology of this observation is also not immediately clear.

Response

We have reviewed our statement to weaken our point. "For both up- and down-regulated genes, the negative suppression score obtained suggests that the hypoxia response program **could** repress flg22 transcriptional response (Fig. 3I). The lower suppression score obtained for down-regulated genes (-0.16) suggests that the repression of down-regulated genes by hypoxia could be of particular importance." However, any other mention of this data in this paragraph is already only stating that the data "suggest".

Line 350: Authors conclude that the erfvii mutant was more severely affected by the combined stress compared to HM and NF conditions - however based on the statistical

differences displayed in figure 4A this is only true for HM. Similar to erfvii, the Col-0 response to NF is also significantly different to the HF condition.

Response

Our conclusions on the stronger effect of the HF treatment in the *erfVII* mutant compared to Col-0 was drawn based on the comparison between HF and HM treatments. A comparison with HM was preferred, because hypoxia had a stronger negative effect on root elongation than flg22 treatment alone, so we reasoned that differences between HF and HM would be most meaningful in order to determine whether the combined treatment had an effect on Col-0 and *erfVII* backgrounds and whether there were differences between the 2 genotypes.

While root elongation is more strongly impacted in both *erfVII* and Col-0 under HF compared to NF, the root elongation in Col-0 was not statistically different under HF compared to HM. In contrast, the *erfVII* mutant's root elongation was more severely affected by HF compared to HM and the difference is statistically significant. Based on this analysis, we conclude that the *erfVII* mutant is more severely affect by HF than by HM alone.

We have amended this section of the manuscript to increase the clarity of our reasoning and conclusion as outlined below:

"Either flg22 (NF) or hypoxia (HM) treatments resulted in decreased root elongation compared to the same genotype in control conditions (normoxia/mock (NM)), with hypoxia having a stronger inhibitory effect than flg22. In Col-0, the combined hypoxia/flg22 (HF) treatment resulted in roots that were shorter than those of seedlings treated with flg22 alone. Compared to hypoxia treatment alone (HM), combined hypoxia/flg22 treatment did not impair further root elongation of Col-0 seedlings. In contrast, the *erfVII* quintuple mutant was more severely affected by the combined HF stress compared to both HM and NF conditions, thus suggesting that ERF-VIIs in the wild type may contribute to protecting the root meristem under combined hypoxia/flg22 treatment."

Line 411-416: Given that it's the title of this paper, could the authors comment on the biological significance of the repression of PTI by hypoxia in the discussion? Intuitively this might be to do with severity of each stress respectively, i.e hypoxia takes 'priority' as this can kill the plant much faster than any pathogen might. 304 genes no longer being differentially expressed is certainly a remarkable observation.

Response

We agree with the reviewer's hypothesis and we believe that this prioritization of hypoxia response over flg22 response is also likely the consequence of resource limitations. The latter is supported by the fact that the novel interaction of HF results in the repression of developmental processes, probably because of the need to balance resource allocation. We have now added the following sentence "One possible explanation is that plants may experience resource limitations that result in the prioritization of their

transcriptional response to hypoxia compared to the onset of PTI, perhaps because hypoxia could have a more immediate impact on plant survival.”.

Response to list of requested changes below:
All comments made below were addressed in the revised version of the manuscript, apart from comment for line 231, because PRR had been defined earlier in the text, as outlined below.

- ☑ Line 35: degradation accumulation
- ☑ Line 122: "suggested a transcriptional link between hypoxia responses and PTI.
- ☑ Line 170: ATE1 should be italicised
- ☑ Line 189: SAG21 should be italicised
- ☑ Line 208: Our data suggest a potential connection between hypoxia and flg22 responses. The RNA-seq analysis also revealed...
- ☑ Line 214: in across
- ☑ Line 219: or and
- ☑ Line 229: (e.g. LBD41 or HRE2; Fig. S1E)
- ☑ Line 230: or and
- ☑ Line 231: Define PRR, I didn't see it beforehand - We had defined PRR in line 116 in the Introduction
- ☑ Line 332: aspects of the flg22 response.
- ☑ Line 333: i.e these genes which no longer
- ☑ Line 337: Not sure if I missed it, define EFR
- ☑ Line 346: Either Both flg22 (NF) or and hypoxia...
- ☑ Line 435-442: Please italicise all mentions of WRKY22 when the gene is being referred to and not the protein.
- ☑ Figure 4B: Italicise gene names (EFR, FLS2, MYB51, CYP83B1) . Done and also corrected in other figure panels elsewhere.

17-Jun-2024

Dr. Emmanuelle Graciet
Maynooth University Department of Biology
Kildare
Ireland

RE: Repression of pattern-triggered immune responses by hypoxia in Arabidopsis

Dear Dr. Emmanuelle Graciet:

We are pleased to accept your manuscript for publication in the Plant Physiology. This acceptance is contingent on revision based on the comments of our reviewers. In particular, please consider the following:

Just one more small hurdle, Emmanuelle! Please correct. I won't send out the ms for review anymore, just want to give you chance to correct this.

Please highlight all changes and include a detailed annotation to changes to the text, with line numbers, and noting your responses to the comments.

To submit your revised manuscript, click:

Link Not Available

If you cannot return the revised manuscript within 8 weeks of receipt, please let us know. Otherwise, we will assume that you have elected not to revise the manuscript and are withdrawing it.

Thank you for allowing us to review your work. We look forward to hearing from you soon.

Sincerely,

Editor, Plant Physiology

-- Reviewer comments:
Reviewer #1 (Comments for the Author):

My questions have been addressed or dicussed. I have a very minor point that "Arabidopsis" in the title should be italic.

Reviewer #4 (Comments for the Author):

Thank you for addressing my previous comments. I have one final point to make based on my previous comment around the conclusions from figure 4A, and the authors response.

Line 387: "In contrast, the erfVII quintuple mutant was more severely affected by the combined HF stress compared to both HM and NF conditions, thus suggesting that ERF-VIIs in the wild type may contribute to protecting the root meristem under combined hypoxia/flg22 treatment"

I apologise if I did not make my previous comment clear. The statistics presented in figure 4a do not support the conclusion that the erfvii quintuple mutant is more severely affected (than Col-0) when we consider both the HF vs HM, and HF vs NF conditions. For both genotypes, in the NF condition, the CLD is 'B'. For 'HF' they are 'CD' and 'D' respectively. The proximity of the letters (e.g. CD being closer to B) does not imply anything about the magnitude or strength of the response, or differences in response between Col0 and erfvii, when we consider HF and NF.

Based on these data and the outcome of the statistical test performed, the erfvii mutant is more severely affected by the combined HF stress compared to the HM condition only. To reflect this, the passage should read 'In contrast, the erfVII quintuple mutant was more severely affected by the combined HF stress compared to the HM condition thus suggesting that ERF-VIIs in the wild type may contribute to protecting the root meristem under combined hypoxia/flg22 treatment"'.

Reviewer #1 (Comments for the Author):

My questions have been addressed or dicussed. I have a very minor point that "Arabidopsis" in the title should be italic.
> Corrected as requested

Reviewer #4 (Comments for the Author):

Thank you for addressing my previous comments. I have one final point to make based on my previous comment around the conclusions from figure 4A, and the authors response.

Line 387: "In contrast, the erfVII quintuple mutant was more severely affected by the combined HF stress compared to both HM and NF conditions, thus suggesting that ERF-VIIs in the wild type may contribute to protecting the root meristem under combined hypoxia/flg22 treatment"

I apologise if I did not make my previous comment clear. The statistics presented in figure 4a do not support the conclusion that the erfvii quintuple mutant is more severely affected (than Col-0) when we consider both the HF vs HM, and HF vs NF conditions. For both genotypes, in the NF condition, the CLD is 'B'. For 'HF' they are 'CD' and 'D' respectively. The proximity of the letters (e.g. CD being closer to B) does not imply anything about the magnitude or strength of the response, or differences in response between Col0 and erfvii, when we consider HF and NF.

Based on these data and the outcome of the statistical test performed, the erfvii mutant is more severely affected by the combined HF stress compared to the HM condition only. To reflect this, the passage should read 'In contrast, the erfVII quintuple mutant was more severely affected by the combined HF stress compared to the HM condition thus suggesting that ERF-VIIs in the wild type may contribute to protecting the root meristem under combined hypoxia/flg22 treatment"'.

> We have corrected the sentence as requested by the reviewer, as it does not alter the main conclusion drawn from this figure, and we realize that our sentence was ambiguous as to which genotype we were referring to in our comparison. Our point was that based on the results of the statistical tests, the *erfVII* mutant was behaving differently under HF compared to the *erfVII* seedlings under HM or NF. Indeed, the CLD letters are different for all of these comparisons:
erfVII NF = B
erfVII HM = C
erfVII HF = D

13-Jul-2024
Dr. Emmanuelle Graciet
Maynooth University Department of Biology
Kildare
Ireland

MSID: PP2024-RA-00593DR2
MS TITLE: Repression of pattern-triggered immune responses by hypoxia in Arabidopsis

Dear Dr. Emmanuelle Graciet:

I am pleased to inform you that your revised manuscript "Repression of pattern-triggered immune responses by hypoxia in Arabidopsis" has been accepted for publication in Plant Physiology. Your manuscript will be checked for consistency with journal formatting and image standards by one of our Science Editors, who will contact you shortly. They may have a number of queries and suggestions for you. Please keep in mind that they work to increase the online visibility of your article and its attraction for readers from the widest of backgrounds. You will have the opportunity to review any revisions, and I hope you will work constructively with them to get the most out of your research. You will also have the opportunity to review all changes when you receive the author proofs for your article.

Once validated for publication, your article will be sent to a Science Editor for a final review of your manuscript and source files. After your final files are uploaded, your article will appear in the next available issue and the online Preview version of your article will be posted after your signed Copyright and Checklist forms and final source files are received.

Please be sure to include the following in your manuscript file:
Harvard style citations
List of all author contributions and funding information at the beginning of manuscript after the titles and author list.
List all supplemental materials associated with the article, with titles, at the end of the ms, before the references and acknowledgments section.
Complete Figure Legends for all figures appearing in the article listed before the Literature Cited.

Thank you and congratulations on your Plant Physiology paper!

Sincerely,
Editor, Plant Physiology

=====
FIRST AUTHOR PROFILES
=====

We recognize the hard work that goes into being First Author of a Plant Physiology paper by publishing a "First Author Feature" for each First Author alongside your paper.

If the first author(s) would like to submit information for a feature on Facebook and Twitter, fill out the form by clicking on the link below:

Plant Physiology First Author Feature Form: https://forms.office.com/r/ZBCGZfhm8F
If you cannot access the form through the link above, please contact Rachel at rbelsky@aspb.org.

To upload an image of yourself to accompany your profile, please put your photo in the correct journal folder at the link below. You must label your image: Author First Name Last Name Article Title.

First Author Profile Image Dropbox: https://www.dropbox.com/scl/fo/xz029p7qjr2jml2rp2kmq/h?dl=0&rlkey=6fkxcaqmxx35ap152b2osy1fz

=====
IMPORTANT REMINDER: PEER REVIEW REPORTS

=====

If you opted to publish a peer review report along with your article during the original submission process, it will be prepared by the editorial staff and publicly posted with your manuscript, inside the zip file that contains any other supplemental material. As a reminder, the peer review report is a public record of all comments from editors and reviewers, as well as your prior responses, as you received them in the decision letters for each draft of your manuscript. If you agreed to publish this report and have changed your mind, or are not sure if you selected this option, please contact the editorial office as soon as possible before signing the license agreement from our publisher.

-- Reviewer comments:
